# Severe delayed autoimmune haemolytic anaemia following artesunate administration in severe malaria: a case report

**DOI:** 10.1186/1475-2875-13-398

**Published:** 2014-10-11

**Authors:** Loic Raffray, Marie-Catherine Receveur, Mathilde Beguet, Pierre Lauroua, Thierry Pistone, Denis Malvy

**Affiliations:** Travel Clinics and Tropical Diseases unit, University Hospital Center of Bordeaux, Bordeaux, France; Centre 897 INSERM & Tropical Diseases unit, CHU de Bordeaux, 1 rue Jean-Burguet, 33 076 Bordeaux, France; Tropical medicine branch, University of Bordeaux, Bordeaux, France; Aquitaine branch of French Blood Institute, Bordeaux, France

**Keywords:** Severe malaria, Artesunate, Autoimmune haemolytic anaemia, Side effects

## Abstract

**Background:**

Parenteral artesunate is recommended as first-line therapy for severe and complicated malaria. Although its efficacy has been proven, long-term safety profile is still under evaluation. Several cases of delayed haemolytic anaemia occurred after initial clinical improvement and resolution of parasitaemia in non-immune travellers and children living in endemic areas. Reports have generated concern that this phenomenon might be related to the treatment itself, either by direct toxicity or immune-related mechanism. This is a report of the first case of autoimmune haemolytic anaemia following treatment of severe malaria initially managed with parenteral artesunate with strong indication for drug-immune related mechanism.

**Case:**

A 17-year old Ivoirian female travelling in France presented with fever, headache and abdominal pain seven days after her arrival. Physical examination was indicative of septic shock while blood analysis showed normal haemoglobin level, but profound thrombocytopaenia and hyperlactataemia. Blood smear analysis showed *Plasmodium falciparum* infection with a parasitaemia of 0.8%. Severe malaria was diagnosed according to the WHO criteria. The patient was initially managed with artemether/lumefantrine combination and then parenteral artesunate for 48 hours. Empiric antibiotic course was also initiated with ceftriaxone, metronidazole, gentamycin, and then piperacillin and ciprofloxacin. At day 14, haemoglobin dropped to 4.6 g/dL with biologic features indicative of haemolysis (LDH 658 U/L, haptoglobin <0.15 g/L). At that time, parasitaemia was negative and other infections or hereditary disorders were excluded, while Coombs’ direct antiglobulin test was positive for IgG and C3d. Antinuclear antibodies were absent. Further investigations evidenced drug-induced antibodies related to artesunate. It was concluded a drug-mediated autoimmune haemolytic anaemia. A corticosteroids regimen was initiated at 1 mg/kg/day. Outcome was favourable and corticosteroids were progressively tapered during two months. At present the patient’s condition remains stable without recurrence of haemolytic anaemia.

**Conclusion:**

This is the first case of delayed haemolytic anaemia related to artesunate with a strong indication for drug-immune related mechanism. Further research is warranted to better characterize this plausible cause of post-treatment haemolysis following parenteral artesunate administration in severe malaria patients.

**Electronic supplementary material:**

The online version of this article (doi:10.1186/1475-2875-13-398) contains supplementary material, which is available to authorized users.

## Background

Infection with *Plasmodium falciparum* malaria remains a major risk for northern countries’ travellers returning from malaria-endemic areas. According to WHO guidelines and recommendations of the European Society for Clinical Microbiology and Infectious Diseases, intravenous (iv) artesunate should be considered as first-line treatment for severe malaria, instead of quinine [1]. While superiority in terms of survival has been proven when iv artesunate was compared to quinine in controlled trials in Asia (SEAQUAMAT) [2] and Africa (AQUAMAT) [3], little evidence is available regarding long-term side effects. Recently, several reports pointed out occurrence of late-onset haemolysis secondary to artesunate administration [4–8]. Most of the cases did not show a clear mechanism underlying this phenomenon, in particular auto-immune mediated processes. Here is reported the first case of auto-immune haemolytic anaemia (AIHA) following treatment of severe malaria initially managed with parenteral artesunate with strong indication for drug-immune related mechanism.

## Case report

A 17-year old Ivorian female without remarkable medical history was admitted for fever, chills, headache, and abdominal pain in a French University Hospital Centre (day 1). She had left Ivory Coast seven days earlier to live in France for studying purpose and symptoms began two days before her admission. Initial physical examination showed a temperature of 39°C and pain when palpating right hypocondrium. Blood tests demonstrated a normal leucocyte count, a thrombocyte count of 11,000/mm^3^ (normal range 150,000-450,000), a haemoglobin level of 12.6 g/dL (12–16) with abnormalities indicative of haemolysis: rise in lactate dehydrogenase (LDH) at 500 U/L (5–248) and total bilirubin at 105 μmol/L (3–18) with a low haptoglobin of 0.15 g/L (0.3-2). She was diagnosed with uncomplicated malaria as peripheral thin blood film showed *P. falciparum* trophozoites (0.8% of parasitized erythrocytes). Abdominal ultrasonography ruled out biliary tract or gall bladder infection. A treatment with oral artemether/lumefantrine combination (Riamet©) was initiated with respectively, 80 and 480 mg trice within the first 24 hours of hospitalization (total of 240 mg of artemether and 1440 mg of lumefantrine). On day 2, her clinical condition deteriorated, her blood pressure dropped to 80/40 mmHg together with a pulse rate of 130 bpm. Laboratory tests indicated a fall of thrombocyte count at 6,000/mm^3^ and elevation of blood lactate up to 7.4 mmol/L (N < 2). The patient was now classified as complicated malaria and admitted to intensive care unit. Treatment was switched to intravenous artesunate (Malacef®, ACE Pharmaceuticals, The Netherlands), started at three doses of 120 mg (2.4 mg/kg body weight) with 12-hour interval. Concurrently she was managed with supportive care, that was administration of 2 l normal saline solution and two units of packed thrombocytes. The use of norepinephrine up to 0.5 μg/kg/min was required during 24 hours to restore a normal blood pressure. The septic shock condition was managed by a course of empiric antibiotic treatment, i.e., ceftriaxone 2 g/day, gentamycin 3 mg/kg/day and metronidazole 1.5 g/day. Evolution was marked by rapid improvement as blood pressure maintained at normal values, and fever disappeared. Lactate level returned within normal range and parasitaemia declined under 0.1% of red blood cells (RBCs). Twelve hours after the last dose of artesunate (total of three doses, 360 mg), the patient was transferred to a regular ward of tropical medicine (day 4). Anti-malarial treatment was continued orally with two daily doses of 80 mg artemether and 480 mg lumefantrine until day 6 (total of 480 mg of artemether and 2880 mg of lumefantrine). Antibiotics were changed to piperacillin/tazobactam (12 g daily) and ciprofloxacin 500 mgx2/day. Despite initial favourable evolution and total clearance of parasites, fever reappeared on day 8. Of note, a drop in haemoglobin up to 6.3 g/dL was evidenced while thrombocytosis was associated: 489 G/L. The Coombs’ test performed at that time was negative. The patient left hospital against medical advice but was re-admitted on day 14 because of persistent fever and marked asthaenia.

**Figure 1:**
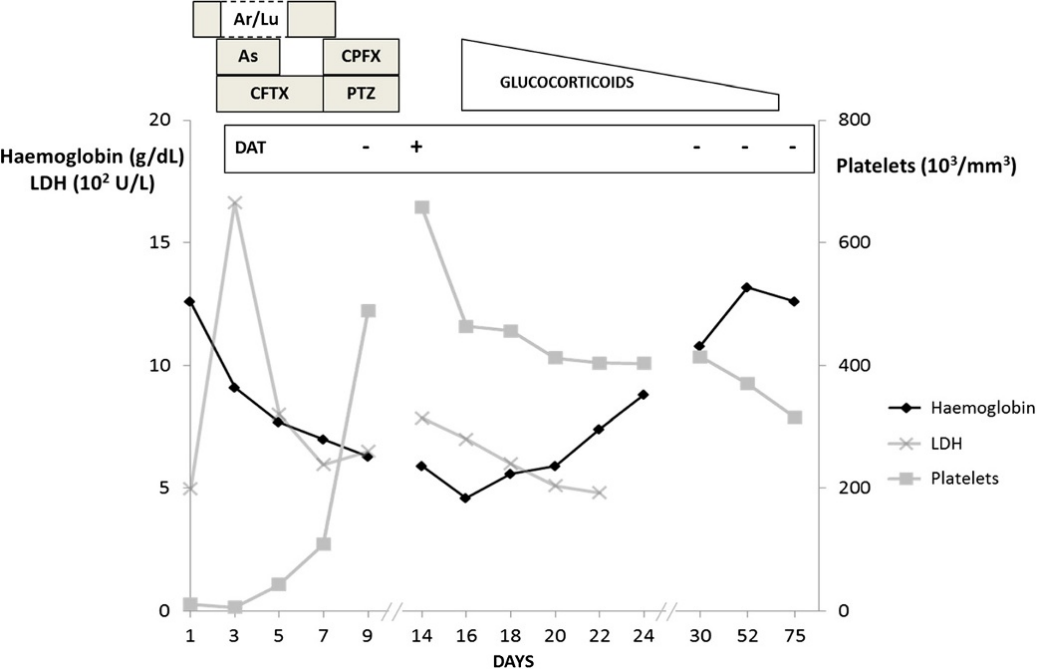
**Evolution of an auto-immune haemolytic anaemia developed during severe malaria treated with intravenous artesunate and other antimicrobial chemotherapy.** Ar = artemether; As = artesunate; CFTX = ceftriaxone; CPFX = ciprofloxacin; DAT = direct antiglobulin test; LDH = lactate dehydrogenase; Lu = lumefantrine; PTZ = piperacillin and tazobactam

Blood analysis confirmed anaemia with a decrease in haemoglobin at 4.6 g/dL and the subsequent criteria for haemolysis: reticulocyte count of 202,000/mm^3^, LDH flare up to 658 U/L and undetectable haptoglobin level. Repeated blood films for malaria as well as for schistocytes (i.e. fragments of RBCs produced by extrinsic mechanical damage within the circulation) were negative. Glucose-6-phosphate dehydrogenase (G6PD) deficiency was promptly ruled out and haemoglobin electrophoresis did not reveal any abnormality. On the other hand, direct Coombs’ test revealed positivity for both IgG and complement factor C3d while this test was negative five days earlier. The irregular antibody testing was negative. It was concluded AIHA and complementary aetiological inquiry was performed. Antinuclear antibodies were absent and the investigation was negative for numerous pathogens such as bacteria (*Mycoplasma pneumoniae, Chlamydia pneumoniae, Salmonella typhi* and *Salmonella paratyphi*), viruses (dengue virus, CMV, EBV, parvovirus B19, HBV, HCV, and HIV) or parasites (*Leishmania spp, Entamoeba histolytica)*.In the context of unusual immunohaematological haemolysis, no transfusion was attempted and corticosteroids were introduced. Methylprednisolone pulses of 60 mg per day during seven days were performed. The haemoglobin level gradually improved (Figure 1). On discharge (day 24), haemoglobin was 8.8 g/dL and treatment was continued with 50 mg of oral prednisone per day. The corticosteroids dosage was progressively tapered during the two following months before discontinuation. Haemoglobin level slowly increased and from day 52 it was measured above the 12 g/dl threshold. At that time haptoglobin and LDH were within normal limits. Consistently, direct Coombs’ test was negative, both for IgG and C3d. During the follow-up, the patient completely recovered and her condition remained stable 12 months later.

Concurrently, she was additionally tested for drug-dependent antibodies by *ex vivo* antigen testing. Serum samples were screened during the convalescent phase, i.e. 12 months, as no biological sample of the acute phase could be retrieved. The test used the main suspected medication substrates, namely ceftriaxone, and three different pharmaceutics specialties containing derivative of artemisin: Riamet®, artemether alone and Malacef (artesunate). This testing was conducted according to a home-made method developed by the French Blood Institute according to standardized reports [9–11] and detailed in the method section.

Among all tested drugs, the test was positive only with artesunate in the papain-pretreatment condition (Table 1). Thus, no other drug could induce haemolysis, even with papain-treated RBCs. This positivity was obtained using two distinct procedures, applied with the three required concentrations of artesunate. This was indicative of the persistence of drug antibodies related to artesunate, even one year after the episode of haemolytic anaemia.

**Table 1:** **Testing for drug-dependent antibodies during convalescent period**

	Patient serum samples					Healthy donor sera		Positive control
	As	Ar	Ar/Lu	CFTX	saline	As	saline	anti-Fya antibody
IAT IgG	0	0	0	0	0	0	0	**++**
IAT C3d	0	0	0	0	0	0	0	
Papain pretreatment	**+**	0	0	0	0	0	0	

Ar = artemether; As = artesunate; CFTX = ceftriaxone; IAT = indirect antiglobulin test; Lu = lumefantrine.

## Methods

The patient’s samples (15 mL of ethylene diamine tetra-acetate blood and 15 mL of serum) were referred to the French Blood Institute of Bordeaux. Sera of healthy individuals of AB blood group that willingly give their blood to the French Blood Institute served as negative controls and were referred as healthy donors. The initial antibody identification was performed using an IAT (anti-IgG and anti-C3d) tube method, with native and papain-treated RBCs (French National Reference Center for Blood Group typing -CNRGS, France). Papain is an enzyme that potentiates the agglutination reaction by reducing the negative charges on the surface of RBCs, thus allowing greater accessibility of some epitopes. Papain treatment of the tested RBCs was performed according to the manufacturer’s recommendations (Papain Palerm, Diagast, Loos, France): one volume of papain solution was added to one volume of washed RBCs. After an incubation of 15 min at 37°C, RBCs were washed three times. Serum samples of the patient were incubated during one hour at 37°C with a panel of erythrocyte and various concentrations of the drugs mentioned above: pure, diluted to 1/10, and estimated therapeutic concentration. Then, agglutination of RBCs was tested with indirect antiglobulin test using anti-IgG or anti-C3d as well as papain pretreated erythrocytes. As negative controls, saline solution and the complement source (pooled, fresh serum of healthy donors of AB blood group) were both tested with and without the drug solution added to reagent RBCs. The positive control referred to erythrocytes incubated with a serum-containing antibody directed against a cell surface protein of RBCs, namely Fya. The result of the test was estimated by the degree of RBCs agglutination with a macroscopic evaluation. If RBCs were not sensitized by antibodies, they were not stuck together and were found at the bottom of the tube (reaction scored ‘0’). If RBCs were sensitized by antibodies, they would remain clustered at the surface column and this reaction was graded from 1 to 4+, 4+ being the strongest positive reaction.

## Discussion

To date, up to 19 cases of putative artesunate-related, late-onset haemolysis have been described in the literature among patients with severe malaria who returned from malaria-endemic regions [4–8]. All patients were non-immune hyperparasitaemic (>5%) travellers from northern countries and haemolysis occurred one to four weeks after parenteral use of artesunate. All patients recovered completely although haemolysis decreased slowly. Concurrently, delayed haemolysis has been recently reported in five among 72 hyperparasitaemic African children, recruited in Gabon or Ghana, and treated with parenteral artesunate for severe malaria [12]. Thus a total of 24 cases of unusual haemolytic anaemia following intravenous artesunate administration have been reported so far. The aetiology of this complication is still unknown.

The fact that mainly hyperparasitaemic patients developed this condition has been related by some to a mechanism called ‘pitting’. After extraction of blood stage parasites during splenic passage, these once-infected erythrocytes have a reduced lifespan compared to naïve erythrocytes with a mean lifespan of around 180 hours and with a total removal of pitted erythrocytes after 28 days. A hypothesis is that haemolytic activity may increase two weeks after acute malaria due to synchronized destruction of pitted erythrocytes [13, 14]. Of note, the patient described here did not show hyperparasitaemia at any time of her history. Besides she originated from West Africa and her presumed semi-immune profile might refer to the initial parasitaemia characterized by a low load level.

Haemolytic anaemia is a challenging situation for physicians as multiple causes may be involved [15, 16]. The characteristic laboratory features are reticulocytosis, increase of unconjugated bilirubin and lactate dehydrogenase, as well as decreased haptoglobin levels. The patient described here presented with these typical abnormalities at first and second admittance. If it is a certainty that malaria was responsible of haemolytic anaemia during the first week, the negative finding for blood smear ruled out its implication in the re-appearance of haemolysis. When there is no obvious aetiology for that kind of anaemia, it is classical to distinguish hereditary (corpuscular) and acquired causes of haemolysis. The former comprises haemoglobinopathies (such as thalassaemia and sickle cell disease), membranopathies (e.g., hereditary spherocytosis) and enzymopathies, such as G6PD deficiency. Blood smear is mandatory to assess some of these causes, and must be completed if necessary by electrophoresis of haemoglobin and/or measurement of G6PD activity. Here, all these tests were negative and the inquiry focused on the process surrounding this acquired haemolytic anaemia: infection, microangiopathy or immune-mediated process. Concerning infectious diseases, relapsing malaria and babesiosis were ruled out, while the lack of schistocytes and thrombocytopaenia argued against microangiopathy. Finally, an immune mechanism was evoked and assessed by a positive direct antiglobulin (Coomb’s) test which is highly sensitive and relatively specific [17, 18]. As the diagnosis of immune-mediated anaemia was confirmed, it had to be classified as auto-immune, allo-immune or drug-induced. Allo-immune haemolytic anaemia could easily be excluded because the patient did not receive RBC transfusion and irregular antibody testing was negative. Concerning AIHA, researches did not show an underlying condition (secondary AIHA) such as a connective tissue disease, a lymphoproliferative disorder or an infection, especially *Mycoplasma pneumoniae* or Epstein Barr Virus-related-mononucleosis. It seems that malaria itself has never been described as a potential cause of AIHA. Although most cases of AIHA are idiopathic, the context of multiple drugs intake and recovery within a few weeks were in favour of a drug-induced immune haemolytic anaemia (DIIHA). Indeed, when a drug responsible for DIIHA is discontinued, the haemolytic anaemia resolves soon afterwards, while during idiopathic AIHA evolution is often chronic or recurrent. In the present case, all suspected treatments were stopped approximately at the same time, what does not permit to charge one in particular. Of note, the patient did not receive artemisin-derivative for previous malaria fever.

Immune-mediated haemolysis may be another mechanism responsible of artesunate-related delayed haemolytic anaemia. In the cases of DIIHA, the direct Coombs’ test (DAT) is usually positive, thus being a prerequisite for hypothesize DIIHA [10]. Thus, among reported cases of artesunate-related delayed haemolytic anaemia, four indicated positivity of Coombs’ test among 12 patients tested [4–6] (Additional file 1). One of these cases showed positivity of indirect antiglobulin test (IAT) consecutive to allo-immunization following previous transfusion [6]. Moreover, IAT was negative in three patients who experienced late-onset haemolysis following artesunate therapy, including one patient with documented negative Coombs’ test [4]. To date, the case reported here is the first describing delayed occurrence of auto-immune anaemia in severe malaria treated with parenteral artesunate with strong indication for drug immune-related contribution.

DIIHA is a rare condition, as the estimated incidence is of one per million population per year. Three mechanisms are described: drug absorption (hapten-induced), immune complex formation with the drug on RBC surface or auto-antibody production resulting in IgG and/or IgM (C3d complement) positivity in direct antiglobulin test [10]. Numerous medications can induce production of antibodies against RBCs, thus positive DAT and IAT, and most of these conditions are clinically and serologically indistinct from AIHA [10, 19]. The commonest drugs involved are anti-infective treatments, especially penicillin and cephalosporins, non-steroidal anti-inflammatory and anti-neoplastics. Given that in the reported case, the patient received several drugs during the week before positive diagnosis of AIHA, it is difficult to assess which one is responsible. Ceftriaxone has been frequently recognized as a cause for DIIHA [20–22], mainly in children who had previously received the antibiotic, unlike in this case. Piperacillin- and ciprofloxacin-induced IHA have seldom been described [10, 19] and, owing to its rarity, these drugs were not screened during IAT of convalescent phase.

Concerning lumefantrine, some cases of haemolytic anaemia are reported [23]. However, most of them can be considered blackwater fever, a syndrome consecutive to anti-malarials of amino-alcohol group and characterized by severe intravascular haemolysis, haemoglobinuria, acute renal failure, occurring immediately after treatment beyond an immuno-allergic pattern. On the other hand, only one case of IHA with lumefantrine was retrieved [24]. In the present case, the patient did not present the clinical and serological findings classically described for DIIHA secondary to ceftriaxone, piperacillin or lumefantrine. Thus it can be hypothesized that the immune-mediated haemolytic anaemia experienced by the patient could be attributable to the use of artesunate. One year after recovering, tests were conducted for screening *in vitro* induction of haemolysis with several of the employed drugs. Usually these tests can show immune-mediated haemolysis dependent of the presence of the medication, indicative of drug-dependent antibodies, but they are not performed routinely by most of the laboratories. The results of these tests were indicative of an artesunate-mediated auto-immune haemolysis. Of note, the positive result was evidenced using papain pretreated RBCs, which is considered to enhance the sensitivity for detection of antibody response challenges, as described elsewhere [25]. Such results were not evidenced with the other derivatives of artemisin-based regimens administered to the patient. Of note, even if papain is aimed to increase sensitivity, the result from our analysis definitely argues for the involvement of artesunate.

Whether this suspected immune-mediated haemolysis is a consequence of artesunate itself or of its excipients is questionable. Indeed, no case of artesunate-associated haemolysis were reported in USA, where the medication is produced by the Army Medical Material Development Activity, while in other countries the manufacturing process is different [26].

Finally, outcome was slowly favourable while using glucocorticoids, although this may be coincidental. Management of DIIHA requires discontinuing the suspected medication: it is often the only treatment. Actually, efficacy of steroids is uncertain because data are limited to case reports and the discontinuation of the drug at the same time is a confounding factor.

## Conclusion

Delayed haemolysis is a frequent and relevant complication in patients treated with parenteral artesunate for severe malaria. The aetiology of this phenomenon is still unknown but the drug may act as substrate for autoimmune mechanism. Patients undergoing this drug should be closely monitored with prolonged follow-up including, in patients with delayed haemolysis, a specific immunological investigation.

## Consent

Written informed consent was obtained from the patient for publication of this Case Report and any accompanying images. A copy of the written consent is available for review by the Editor-in-Chief of this journal.

## Electronic supplementary material

Additional file 1: **Cases reported in the literature of delayed haemolytic anaemia with positive Coombs’ test after intravenous artesunate therapy for severe malaria.** (DOCX 14 KB)

## Abbreviations

AIHA: Autoimmune hemolytic anemia
DIIHA: Drug-induced immune haemolytic anaemia
G6PD: Glucose-6-phosphate dehydrogenase
IAT: Indirect antiglobulin test
LDH: Lactate dehydrogenase
RBC: Red blood cell.
