# Supplementary material for: Severe delayed autoimmune haemolytic anaemia following artesunate administration in severe malaria: a case report
Source: Malar J. 2014 Oct 11;13:398. doi: 10.1186/1475-2875-13-398 (PMC4203878; doi:10.1186/1475-2875-13-398)
Supplement: Supplementary file 1 — Additional file 1: Cases reported in the literature of delayed haemolytic anaemia with positive Coombs’ test after intravenous artesunate therapy for severe malaria. (DOCX 14 KB) [file 12936_2014_3562_MOESM1_ESM.docx]

Additional file 1: Cases reported in the literature of delayed haemolytic anaemia with positive Coombs test after intravenous artesunate therapy for severe malaria

| Case | Source | location | Brief description | Peak parasitaemia | Initial treatment | Cumulative dose of artesunate | PCT (days) | Initial Hb (g/dL) | Lowest Hb  (g/dL) | Time to nadir (days) | Results of Coombs’ test | Drug-dependant antibodies test | Treatment for hemolysis |
| --- | --- | --- | --- | --- | --- | --- | --- | --- | --- | --- | --- | --- | --- |
| 1 | Ref [5] | Belgium/  Netherlands | Male of 53 years with impaired consciousness | 34% | quinine | NA | 4 | 12.9 | 6.9 | 20 | C3d+ | not performed | none |
| 2 | Ref [5] | Belgium/  Netherlands | Female of 44 years | 37% | quinine | NA | 4 | 9.7 | 6.1 | 15 | IgG+ and C3d+ | not performed | transfusion and corticosteroids |
| 3 | Ref [5] | Belgium/  Netherlands | Female of 50 years with haemoglobinuria | 30% | artesunate | NA | 10 | 11.6 | 6.9 | 13 | IgG+ and IgM+ | not performed | transfusion and corticosteroids |
| 4 | Ref [6] | Germany | Male of 54 years with septic shock, impaired consciousness and acute renal failure | 21% | artesunate | 9 mg/kg | 7 | NA | 5.7 | 14 | IgG+  (anti-E specificity) | not performed | transfusion |
| 5 | Our case | France | Female of 17 years with septic shock | 0.8% | artemether lumefantrine | 16.2 mg/kg | 3 | 12.6 | 4.6 | 14 | IgG+ and C3d+ | positive for artesunate | corticosteroids |

Abbreviations: Hb=haemoglobin ; NA=not available; PCT=parasite clearance time
